# A detailed open access model of the PubMed literature

**DOI:** 10.1038/s41597-020-00749-y

**Published:** 2020-11-20

**Authors:** Kevin W. Boyack, Caleb Smith, Richard Klavans

**Affiliations:** 1 SciTech Strategies, Inc., Albuquerque, NM USA; 2 University of Michigan Medical School, Ann Arbor, MI USA; 3 SciTech Strategies, Inc., Wayne, PA USA

**Keywords:** Decision making, Research management, Translational research, Funding, Publication characteristics

## Abstract

Portfolio analysis is a fundamental practice of organizational leadership and is a necessary precursor of strategic planning. Successful application requires a highly detailed model of research options. We have constructed a model, the first of its kind, that accurately characterizes these options for the biomedical literature. The model comprises over 18 million PubMed documents from 1996–2019. Document relatedness was measured using a hybrid citation analysis + text similarity approach. The resulting 606.6 million document-to-document links were used to create 28,743 document clusters and an associated visual map. Clusters are characterized using metadata (e.g., phrases, MeSH) and over 20 indicators (e.g., funding, patent activity). The map and cluster-level data are embedded in Tableau to provide an interactive model enabling in-depth exploration of a research portfolio. Two example usage cases are provided, one to identify specific research opportunities related to coronavirus, and the second to identify research strengths of a large cohort of African American and Native American researchers at the University of Michigan Medical School.

## Abstract (summary)

**Table:** 

Measurement(s)	Biomedical Research • document relatedness
Technology Type(s)	digital curation • machine learning • Cluster Analysis • text similarity • citation analysis

Machine-accessible metadata file describing the reported data: 10.6084/m9.figshare.13010537

## Background & Summary

Portfolio analysis is a common practice in the finance world where options (e.g., stocks, bonds) are well defined. Portfolio analysis is also being increasingly done in research institutions. It is a more difficult problem here, however, because research options – the topics of research – are not rigorously defined. Research administrators thus often have a somewhat cloudy view of their institution’s research activity which means that their visions and missions are difficult to translate into plans involving concrete choices. Due to ambiguity in the definition of research options, evaluation of potential responses to societal and economic pressures are likewise ambiguous.

Fuzzy descriptions of research options are now being replaced by highly detailed and accurate models of the scientific literature. For instance, tens of millions of documents in the Scopus database have been grouped into 91,000 document clusters using extended direct citation among documents^1^, a process that has been shown to create coherent clusters^2–4^. This same process was replicated and made available in Elsevier’s SciVal product where document clusters (called “Topics”) are now used by many institutions for portfolio analysis and research decision-making^5^. The most recent versions of the Leiden Ranking^6^, developed by the Centre for Science and Technology Studies (CWTS) at Leiden University, are based on a model of science that consists of 4,535 document clusters^7^ (referred to as micro-level fields) partitioned from the citation network. While these previous works made use of subscription-based citation databases (Scopus and the Web of Science), the goal of this work was to create a similarly accurate model based on the (openly available) PubMed literature for strategic decision-making in biomedical research.

**Fig. 1:**
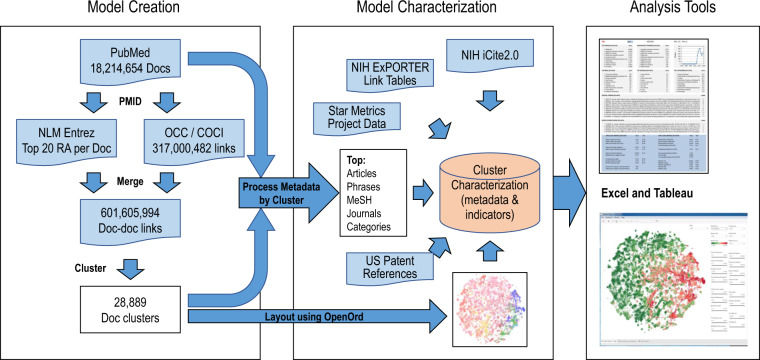
Data and process used to create the PubMed model and associated tools.

Figure 1 illustrates the process used to create the model along with the resulting framework. The first major step is the creation of a detailed model of science. Using over 18 million PubMed records from 1996–2019, each with a PubMed identifier (PMID), we retrieved similar article (SA) scores using an Entrez e-utility and citation links from the OCC^8^ and COCI^9^ databases. After merging, we used the resulting 606.6 million document-document links to cluster the documents into 28,889 clusters using the Leiden algorithm^10^. The second major step is to characterize each cluster using the document level metadata along with US patent reference data, US National Institutes of Health (NIH) and National Science Foundation (NSF) project data from Star Metrics, paper-to-project link tables from NIH ExPORTER, and additional metrics from the NIH iCite2.0 database^11,12^. We also created a visual map of the clusters using the OpenOrd layout routine^13^ and cluster-level relatedness. Some clusters were removed from the model at this point. Finally, we loaded the resulting cluster-level metadata, indicators and cluster positions into Excel and Tableau workbooks; the Excel workbook makes the data readily available for re-use while the Tableau interface enables visual exploration and filtering of the model for detailed analysis.

This model is the first highly granular characterization of the PubMed literature that can be used for portfolio analysis at the level of research topics. It is also the first large scale model of any literature that is based on both citation links and the textual relatedness of documents and is thus among the most accurate characterizations of the literature ever created. The open database and tool contain detailed information that can be used to search and explore topics related to biomedical science, and to analyze these topics within the context of funding, industrial application, clinical application, translational potential, and other features.

In addition, this model is complementary to the recently published PubMed Knowledge Graph (PKG)^14^ which contains document level information from PubMed and other sources such as extracted bioentities, disambiguated authors and institutions. The cluster-level analytics enabled by our model are an important addition to the type of data provided by PKG, enabling both macro- and micro-level analysis of the research landscape.

## Methods

The methodology used to create and characterize our PubMed model uses and combines data from many sources as listed in Table 1.

**Table 1:** Primary data sources, sizes and brief descriptions, 1996–2019.

Data Source and Version	# Records	# PMID	Description
PubMed, pubmed.ncbi.nlm.nih.gov	18,214,654	18,214,654	Bibliographic metadata
NIH iCite2.0^15^ (Jan 2020)	18,214,654	18,214,654	Paper-level metrics (translation, RCR, etc.)
Open Citation Collection^15^ (Jan 2020)	315,512,095	12,578,393	Citation links by PMID
OpenCitations (Jan 2020), https://opencitations.net/index/coci	186,399,013	7,783,835	Citation links by DOI
PMID Similar Article Scores (top 20 current as of Jan 2020)	364,534,609	18,205,619	Text-based relatedness scores based on Lin & Wilbur^17^, retrieved using Entrez e-utility
Star Metrics (2008–2018), https://federalreporter.nih.gov/FileDownload	861,170	n/a	Annual project data (including funding amounts) for NIH, NSF and other US agencies
NIH ExPORTER (1996–2018), https://exporter.nih.gov/ExPORTER_Catalog.aspx?sid=0&index=5	4,224,360	1,789,416	Link tables – PMID to NIH project
NSF Awards API (1996–2017), https://www.research.gov/common/webapi/awardapisearch-v1.htm	566,155	149,091	List of references by NSF project, matched to PMID
USPTO Non-patent references, 2015–2019, https://bulkdata.uspto.gov/	2,952,584	660,581	Full text XML of US patents, non-patent references were extracted and matched to PMID

For the model we chose to include PubMed documents from 1996–2019. Given that the model is intended to be used for planning and evaluation of recent trends, we felt no need to include historical documents much older than 20 years. 1996 was chosen as the starting year to enable comparison to our Scopus-based models^1^ if need arises. Documents were also limited to those that either had references in the NIH Open Citation Collection (OCC)^8,15^ or for which the National Library of Medicine (NLM) had already calculated similar article (SA) scores, resulting in a set of 18,765,313 documents.

Before calculating relatedness between pairs of papers, we further filtered the documents by removing those from a set of 42 journals, primarily from the physical sciences, that were found in a preliminary study to create clusters that were unconnected to the biomedical core of the document set. These clusters, which contained papers from disciplines such as high energy physics, physical chemistry and crystallography, were distracting to early users of our models since they had nothing to do with biomedicine. Although many other journals could have been removed, we chose those that were large and whose removal would most affect the overall model. Upon removal of the 550,659 documents in these journals, our set was left with 18,214,654 documents, which were then used to create the model.

### Relatedness measure and clustering

Our model is constructed using a hybrid relatedness measure composed of direct citation (DC) and textual SA scores. We use a 50:50 DC + SA hybrid measure that our most recent study found to be more accurate than either a pure citation-based measure or pure text-based measure^16^.

The relatedness *r*_*ij*_ between papers *i* and *j* is calculated as

1 $${r}_{ij}^{HYB}=\alpha \,{r}_{ij}^{DC}+\left(1-\alpha \right){r}_{ij}^{SA}$$

The parameter *α* is set such that *α ∑ r*_*ij*_^*DC*^ = (*1 - α*) *∑ r*_*ij*_^*SA*^ to achieve a 50:50 weighting of citation and textual relatedness across the entire set of document pairs.

2 $${r}_{ij}^{DC}={\rm{\max }}\left({c}_{ij},{c}_{ji}\right)$$

where *c*_*ij*_ = *1/nref* if *i* cites *j* and is 0 if not, and *nref* is the number of references in document *i* within the OCC set, and

3 $${r}_{ij}^{SA}={S}_{ij}/{\rm{\max }}\left({S}_{ij}\right)$$

using SA scores (*S*). Since SA scores are symmetrical, in cases were document pairs *ij* and *ji* were both within the set, only the *ij* pair was included. Each type of relatedness value was normalized to its corresponding maximum. Thus, all values of *r*_*ij*_^*DC*^ and *r*_*ij*_^*SA*^ ranged between 0 and 1.

Citation links for the citation portion of the relatedness measure were obtained from two sources. First, we used the January 2020 version of the OCC linkage set which contained references for 13,013,385 (71.44%) of the documents in our set. Of these, 12,578,393 cited other PMID within the set, comprising 315,512,095 citation links. It is important to use complete data where possible to obtain optimum results. We note that complete reference data are not available from any source, paid or open. Reference data can be missing for several reasons including publishers not making references available to aggregators (such as Scopus or WOS) and lack of open data. For comparison, while the OCC is missing references for 13.1% of PubMed documents in 2017, Scopus is also missing references for 7.4% of the same set of documents. Overall, the OCC is a relatively complete source of reference data (over 80%) for recent years and is thus very suitable for use in science mapping studies.

Second, we used data from OpenCitations (COCI) that were downloaded on January 20, 2020 and converted those data to PMID using DOIs. After matching the OCC and COCI data, it was found that the COCI data contain 186,399,013 links between pairs of PMID within our set, of which only 1,488,388 are not in the OCC linkage set. The COCI set contains references for only 27,065 documents that are not covered in the OCC set. Thus, the OCC data are a relatively complete set on their own and the COCI data add little to the total. The combined OCC/COCI sets of citation links contained 317,000,482 links between PMID within our document set.

For the textual component of the hybrid relatedness measure, we used the similar article (SA) scores (https://www.ncbi.nlm.nih.gov/books/NBK3827/#pubmedhelp.Computation_of_Similar_Articl) calculated by NLM using words from titles, abstracts and MeSH terms^17^, and which can be retrieved using an Entrez e-utility (https://www.ncbi.nlm.nih.gov/books/NBK25499/, see cmd = neighbor_score). We chose to use the top 20 SA scores for each document, limited to those where the paired document was also within the set, resulting in a set of 363,404,050 links of which 314,316,142 were unique; i.e., *S*_*ji*_ and *S*_*ij*_ pairs were not both present. The choice to use the top 20 scores per paper was made because previous research showed that there was little difference in clustering accuracy whether 12 or 40 links per document were used^18^.

Combining the citation and SA links resulted in a set of 601,605,944 links of which 30,560,355 had a direct citation link and a top 20 SA score. This overlap (9.64% of the direct citation links also had a top 20 SA link) is quite low, suggesting that citation-based and text-based relatedness are complementary and that both are ultimately important to accurate clustering. There were 286 million pairs of documents that had a direct citation link but that did not have a top 20 SA score. This does not mean that there is no textual relatedness between these pairs of documents. In most cases there is topical overlap between pairs of documents linked through citation^19^. Rather than assuming no textual relatedness for these pairs, we used SA scores even though they were outside of the top 20. Actual SA scores were used for pairs for which they were available, and estimated scores (half the minimum SA score for either document in the pair) were added where a calculated score was not available. The summed normalized SA and DC scores were *∑ r*_*ij*_^*DC*^ = 9359293 and *∑ r*_*ij*_^*SA*^ = 26297313; solving *α ∑ r*_*ij*_^*DC*^ = (*1 - α*) *∑ r*_*ij*_^*SA*^ gives α = 0.7375 to achieve a 50:50 weighting of citation and textual relatedness. Equation (1) was then used to calculate hybrid relatedness for each document pair. For example, PMID 18637048 cites 26 papers within the set, one of which is PMID 15000003. For this pair of documents *r*_*ij*_^*DC*^ = 0.038462 and *r*_*ij*_^*SA*^ = 0.136923 which leads to *r*_*ij*_^*HYB*^ = 0.064307.

The full list of document-document pairs and their relatedness values (which are used as edge weights) were used to cluster the documents. We desired a model with approximately 30,000 clusters at a minimum size of 75 documents each. This specification is based on previous work where we have found that models with an average cluster size of several hundred documents tend to contain clusters that are sufficiently large to be meaningful and well differentiated to experts^20^ but without being so large as to be about multiple topics. A three-level hierarchical clustering was created using the Leiden algorithm^10^ with input resolutions as indicated in Table 2. Different quality functions can be used with the Leiden algorithm; we used the original quality function introduced in an earlier version of Leiden University’s clustering methodology^21^. The most granular level is designated PM5 to denote that this is a PubMed (PM) model and that the cluster sizes are similar to those of our Scopus model with around 10^5^ (5) clusters. Higher level clusters are also designated by their rough order of magnitude – e.g., PM4 contains on the order of 10^4^ clusters.

**Table 2:** Properties of the three-level PubMed model.

	PM5	PM4	PM3
Resolution	7.75E-05	10	21.25
Minimum cluster size	75	750	7500
# Clusters	28,889	3074	288
Largest cluster	6052	44,163	583,979
Ratio largest::smallest	80.7	56.0	74.0

### Visual map

Once the clustering was completed, a visual map of the clusters was created. Relatedness (*R*) between clusters *m* and *n* was calculated as the summed relatedness values

4 $${R}_{mn}^{HYB}=\sum \,{r}_{ij}^{HYB}/\surd ({N}_{m}{N}_{n})\,(i\,\epsilon \,m,\,j\,\epsilon \,n)$$

where *N*_*m*_ and *N*_*n*_ are the number of documents in clusters *m* and *n*, respectively. The denominator serves to normalize for cluster size so that the relatedness values are not inherently biased toward large clusters.

Cluster-cluster relatedness values were then filtered to the top 15 per cluster, and OpenOrd^13^ was used to create a graph layout of the clusters with the cut parameter set to 0.7. OpenOrd returns [x, y] positions for each cluster on a 2D plane.

**Fig. 2:**
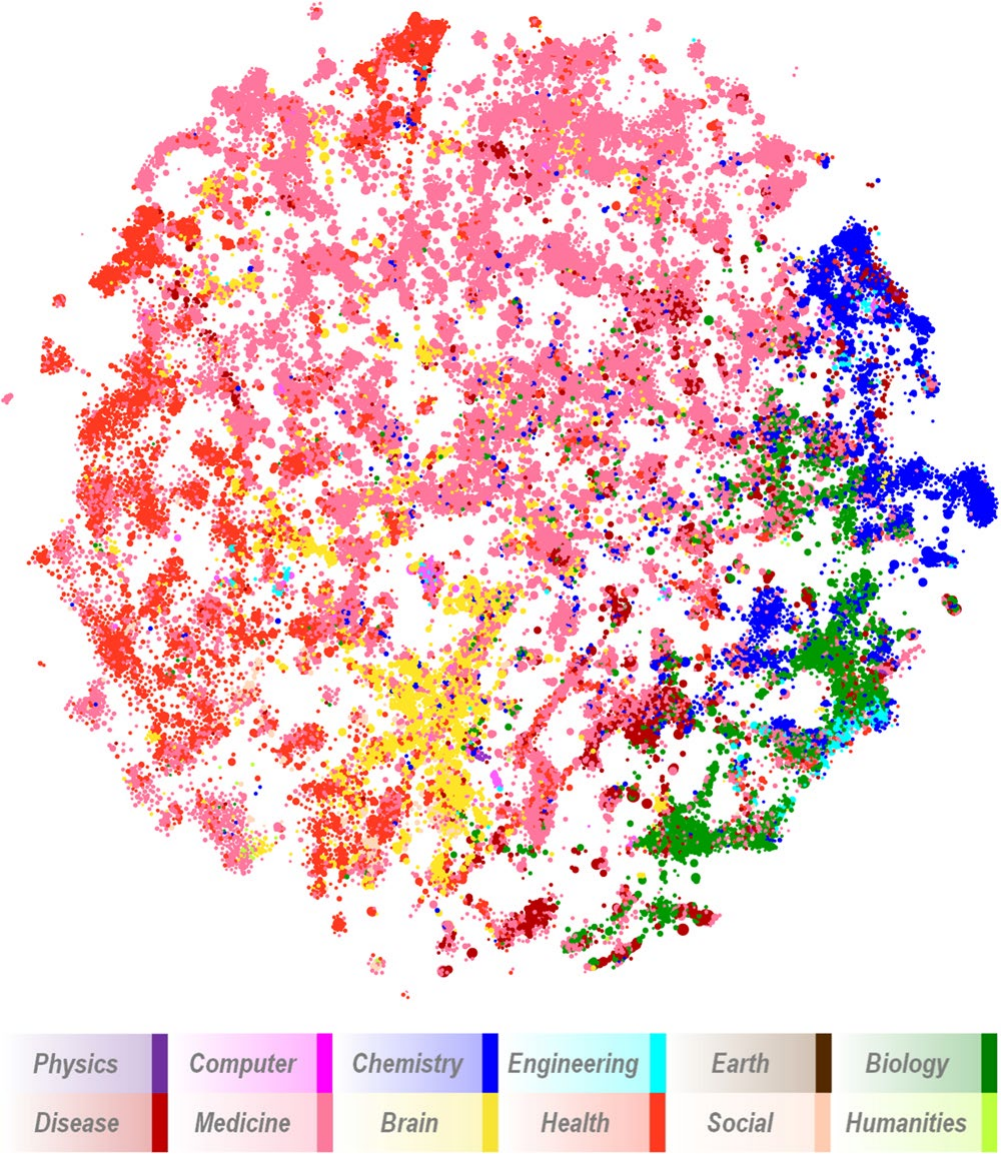
Visual map of the PubMed model showing 28,743 clusters. Each cluster is colored according to its dominant field (see legend).

Each cluster is assigned to one of 12 major fields (e.g., Biology, Medicine, Brain Science, etc.) based on the journal distribution within the cluster^1^ and a journal-to-field mapping previously developed^22^ and the cluster is colored in the map based on its dominant field (see Fig. 2). Clusters that are highly related are close to each other in the map and those that have little or no relationship to each other have more distance between them. A two-dimensional map is simply a visual approximation of a multi-dimensional system. In addition to showing the position of each cluster, the map can be used as a basis for the overlay of other information or to show filtered results.

The high-level layout of the map is similar to that found in other maps of science. For instance, chemistry and biology are proximate, and infectious disease tends to fall between biology and medicine. Health sciences, which mostly comprise clinical areas, nursing, etc., are at the outer edge of the map but also have interfaces with medicine and brain sciences. Relatively few clusters in physics, computer science, engineering, earth sciences, social science and humanities are found in the map.

In our initial exploration of the map we noticed several groups of clusters that were either not connected to the main component of the map or that were dominated by physics. The group of physics clusters was reviewed, and 25 clusters were found to have no biomedical content and manually discarded from the map and model. Investigation of other groups of clusters showed several groups of clusters with no discernible topic focus. In many cases they were clusters of documents with *errata/corrigendum/correction* in their titles, or clusters of documents with no abstracts and few references. 121 of these clusters were also removed manually. The final PM5 map consists of 28,743 clusters containing 18,160,327 documents.

### Model characterization

**Fig. 3:**
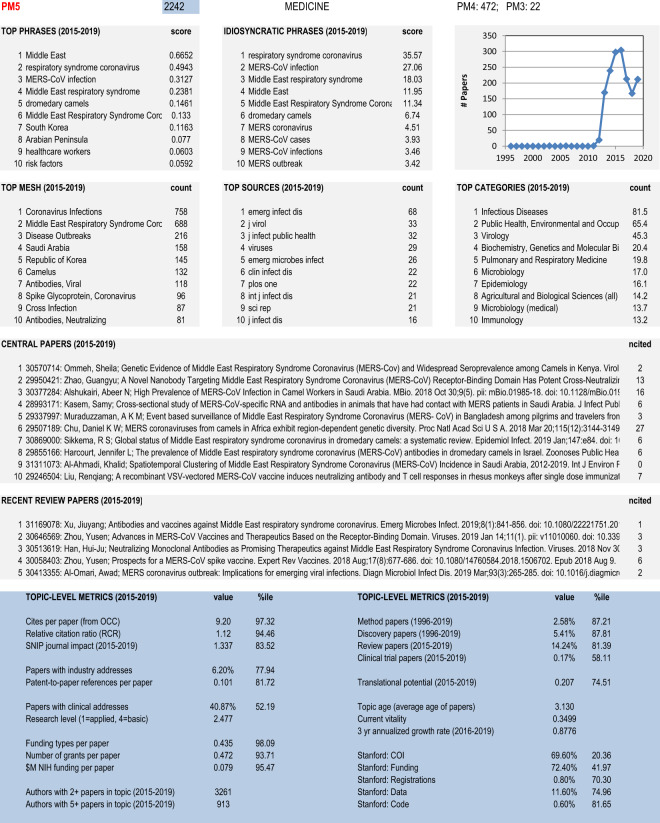
Detailed characterization of a single cluster in the Excel workbook.

A variety of metadata and indicators were used to characterize the clusters in the model to enable practitioners and decision makers to recognize and analyze topic-level structures. Figure 3 shows an example of this characterization for a single cluster. Except for the chart showing the number of documents by year in the upper right corner, characterization was done using only those documents published from 2015–2019 to focus on recent content.

Lists of top ten phrases, idiosyncratic phrases, MeSH terms, sources (journals), and journal categories are provided to characterize the topic embodied by each cluster. Additional nuance is added by listing the ten most central papers and five most central review papers.

Central papers and reviews are chosen by calculating the sum of the relatedness scores *r*_*ij*_^*HYB*^ from Eq. (1) where papers *i* and *j* are in the same cluster and then sorting by summed scores. Those with the highest within-cluster relatedness scores are assumed to be the most central to the cluster.

Phrases are identified using the following method:

1. The NLTK library for Python is used to extract noun phrases (NP) from titles and abstracts using the grammar NP: {<JJ.*|VBG > * < NN.* > + <VBG> ?} which matches a sequence of zero or more adjectives (JJ.*) or gerunds (VBG), followed by one or more nouns (NN.*), followed by 0 or 1 gerund.
2. The process in step 1 is repeated 20 times for each cluster using a random one-third sample of its titles and abstracts. Phrase counts are then summed over the 20 bootstrapped samples. We have noticed that phrase scores obtained from bootstrapping are more well separated than those obtained from simply running over the cluster contents.
3. Counts were then transformed to scores as *sc* = *count*/(20*np/3) where *np* is the number of papers in the cluster (2015–2019). Thus, the score is an estimate of the number of times the phrase occurs per paper in the cluster.

For idiosyncratic phrases, we take the full list of scores from step 3 above, and then re-score them using idio = 10*log(1 + sc/nptot)*sc/sctot, where *sc* is the score from step 3, *sctot* is the sum of scores for that phrase over all clusters, and *nptot* is the total number of papers in all clusters in which the phrase appears. This formula effectively re-ranks phrases by how much they differentiate one cluster from another. If a phrase is common and occurs in many clusters, the score adjusts down in a relative sense and vice versa. Having access to the most common phrases in a cluster and to those that differentiate a cluster from other clusters provides greater perspective on the cluster contents than if only one or the other were reported.

This method for labeling clusters is similar to that of Waltman & van Eck^21^ in some respects, but differs in others. While the two methods use different toolkits, the functions are similar, both focusing on noun phrases with some allowance for adjectives. Also, our method for re-scoring phrases based on their relative prevalence in the cluster and corpus is similar in intent to Waltman’s calculation of term relevance scores.

Top categories uses the Scopus ASJC classification system and is based on the journal-to-ASJC file that is publicly available at the Scopus website^23^. ASJC categories were chosen because the list of categories is larger and seems more intuitive to us than those in either the ScienceMetrix or Australian Research Council journal classification systems.

Metrics are also calculated for each cluster. For many of these the actual metric value is given, and a percentile score is also given, where the percentile is related to the cluster ranking for that metric. For instance, for the cluster shown in Fig. 3, the average cites per paper is 9.20, and this value is in the 97th percentile among all clusters. The metrics reported by category for each cluster include:

- Impact
  - Mean cites per paper from iCite2.0^8^.
  - Mean relative citation ratio (RCR) from iCite2.0^12^. RCR is a metric developed several years ago by NIH that normalizes citation counts of each paper based on its local co-citation network.
  - Mean SNIP journal impact^24^ using the Scopus journal file^23^. SNIP is a journal level impact factor that accounts for differences in citation practices by field.
- Industry involvement
  - Fraction of papers with an industry address. Papers with an industry address were identified using a method similar to that of Tijssen^25^ by searching addresses for abbreviations associated with companies (e.g., Corp., Inc., Ltd., GmbH, BV) and prominent company names (e.g., Merck, Novartis).
  - Mean patent-to-paper citations per paper. We have over many years systematically mined non-patent references from US patents and matched those reference strings to indices built from Scopus and PubMed article data to find likely matches. The resulting matches are used to calculate patent-based metrics.
- Clinical involvement
  - Fraction of papers with a clinical address. Papers with a clinical address were identified by search addresses for strings associated with clinical institutions such as hospitals and medical centers (e.g., spital, clinic, klinik, medical center, cancer center, NHS)^25^.
  - Mean research level on a scale of basic to applied using the method for calculating research level by paper. The machine learning approach used here was trained on titles and abstracts of papers from over 4,000 journals using the journal research levels^26^.
- Funding
  - Mean number of funding types per paper (using PubMed “Research Support” tags).
  - Mean number of grants per paper.
  - Mean funding per paper (in $$M).
- Document type
  - Fraction of papers classified as a *method* paper^27^. Method papers were identified using citing sentences and citing locations from PubMed Central full text. Machine learning was based on a training set of 1000 manually classified papers with the best classifier achieving 92% accuracy.
  - Fraction of papers classified as a *discovery* paper^28^. Discovery papers were identified using specific terms in citing sentences from PubMed Central full text. Machine learning was based on a manually curated set of 135 discovery papers (158 non-discovery papers were excluded) with a classifier accuracy of 94%.
  - Fraction of papers classified as reviews by PubMed.
  - Fraction of papers classified as clinical trials by PubMed.
- Translation
  - Mean translational potential using Approximate Potential to Translate (APT) from iCite2.0^11^. APT is a metric recently developed by NIH based on multiple features including citation patterns between different types of MeSH terms (e.g., papers with *Human* terms citing papers with *Molecular, Cellular and Animal* terms).
- Authorship/community strength
  - Number of authors with at least 2 papers in the cluster.
  - Number of authors with at least 5 papers in the cluster. These metrics are based on the principle that communities (or topics) with many active authors are stronger than those with few.
- Age and momentum
  - Mean age of papers.
  - Current vitality, based on the inverse of mean reference age^29^. This metric is based on the principle that fast growing topics are based on relatively young (rather than old) literature.
  - 3 year annualized growth rate from 2016–2019^29^.
- Transparency indicators extracted from PubMed Central open access (PMCOA) full text articles, 2015–2019^30^. Researchers at Stanford manually identified the below listed types of statements in 500 articles. Machine learning developed methods to identify such statements in other full text papers and was applied to the full PMCOA corpus. Specificity and sensitivity were both above 90%.
  - Fraction of papers that are open access (OA) from PMCOA.
  - Fraction of OA papers with COI statements.
  - Fraction of OA papers with funding statements.
  - Fraction of OA papers with registration statements.
  - Fraction of OA papers with data sharing statements.
  - Fraction of OA papers with code sharing statements.

Cursory explanatory details have been given above for several of the metrics along with associated references that contain more information about the scope and accuracy of the metric. Detail has not been given for metrics that are more common or that are self-explanatory.

Regarding funding information, PubMed contains a set of Research Support tags that specify different types of funding. For example, NIH-intramural, NIH-extramural, US-govt-non-NIH, and non-US-govt are four of the types. These data are used to calculate the mean number of funding types per paper. However, these numbers are questionable as recent research suggests that NIH may be indexing less than half of the acknowledged funding content available in articles^30^. PubMed indexes acknowledged grant numbers from a limited list of funding sources with a heavy emphasis on NIH grants. These data are used to calculate mean number of grants per paper. For funding amounts, grant-to-article links from NIH RePORTER and the US National Science Foundation (NSF) API were used to calculate the numbers of papers by grant per cluster. The funding amount for each grant from the StarMetrics Federal RePORTER data (see Table 1) was fractionally assigned to the clusters containing those papers, summed, and then used to calculate mean funding (from NIH and NSF) per paper.

## Data Records

As explained in the method section, metadata from PubMed records and multiple other sources were used to characterize the 28,743 clusters in our PubMed model. These characterizations comprise a derivative database that is freely available on Figshare under the CC BY 4.0 license in two formats – an Excel workbook and a Tableau workbook^31^. The Excel workbook makes the data readily available for re-use while the Tableau interface enables visual exploration and filtering of the model for detailed analysis.

### Excel workbook

The Excel workbook is comprised of 15 different sheets as shown in Table 3. The majority of the sheets contain cluster-level metadata (e.g., top 10 phrases, top 10 MeSH headings) or metrics. PM5_SHEET allows the user to input a cluster number and then self-populates with data from other sheets to create the characterization shown in Fig. 3. One sheet contains the list of 42 journals that were excluded from our model and one sheet contains the list of PMID identified as method^27^ or discovery^28^ papers which is not available elsewhere. Field names are given in the first row of each sheet. Data across different sheets are linked through the cluster number (PM5).

**Table 3:** Description of sheets in the Excel workbook.

Sheet Name	# of Lines	Description
CLUST	28,743	Cluster positions, metrics and percentiles
TRANSP	28,743	Transparency metrics by cluster using Stanford data extractions from PMCOA documents, 2015–2019
QUERY	28,743	COVID/University query counts by cluster, 2015–2019
COUNT	28,743	Annual document counts by cluster, 1996–2019
PHRASE	280,200	Top 10 phrases by cluster (rank, phrase, score), 2015–2019
IDIO	280,200	Top 10 idiosyncratic (differentiating) phrases by cluster (rank, phrase, score), 2015–2019
MESH	286,296	Top 10 MeSH headings by cluster (rank, MeSH, count), 2015–2019
ASJC	275,606	Top 10 journal categories by cluster (rank, category, count), 2015–2019
JNL	234,438	Top 10 journals/sources by cluster (rank, journal, count), 2015–2019
AUTH	286,425	Top 10 authors by cluster (rank, count, cpp, author), 2015–2019
CORE	284,441	Top 10 most central papers (excluding reviews) by cluster (rank, score, type, bibentry, cites), 2015–2019
REVIEW	109,171	Top 5 most central review papers by cluster (rank, score, type, bibentry, cites), 2015–2019
PM5_SHEET		Enter PM5 cluster number to populate this sheet with metadata from the preceding sheets
JNL_EXCL	42	Journals excluded from the model
METHDISC	764,405	List of method and discovery papers by cluster (PMID, meth, disc), 1996–2019

Descriptions of the data fields for the CLUST and TRANSP tables are provided in Tables 4 and 5, respectively.

**Table 4:** Data types for records in the CLUST Excel sheet.

Index	Format	Description
PM5	Integer	PM5 cluster number
PM4	Integer	Corresponding PM4 cluster number
PM3	Integer	Corresponding PM3 cluster number
X	Double	X coordinate value on map
Y	Double	Y coordinate value on map
field	String	High-level field of science, see Fig. 2 for legend
nptot	Integer	Number of documents, 1996–2019
np1519	Integer	Number of documents, 2015–2019
cpp19	Double	Mean cites per paper for documents 2015–2019 as of end-2019
cpp19_pctl	Double	cpp19 percentile among clusters
rcr	Double	Mean RCR (relative citation ratio) value, 2015–2019
rcr_pctl	Double	rcr19 percentile among clusters
snip	Double	Mean SNIP (source normalized impact factor), 2015–2019, 2018 SNIP value used for 2019 documents
snip_pctl	Double	snip percentile among clusters
apt	Double	Mean APT (approximate potential to translate) value, 2015–2019
apt_pctl	Double	apt percentile among clusters
ind_fr	Double	Fraction of documents with at least one industry affiliation/address, 2015–2019
ind_pctl	Double	ind percentile among clusters
nprpp	Double	Mean number of patent citations per paper, patents 2015–2019, documents 1996–2019
npr_pctl	Double	nprpp percentile among clusters
clin_fr	Double	Fraction of documents with at least one clinical affiliation/address, 2015–2019
clin_pctl	Double	clin percentile among clusters
rlev	Double	Mean research level, 2015–2019
fundpp	Double	Mean number of funding types per paper, 2015–2019
nf_pctl	Double	fundpp percentile among clusters
grantpp	Double	Mean number of grants indexed in PubMed per paper, 2015–2019
ng_pctl	Double	grantpp percentile among clusters
starpp	Double	Mean funding per paper in $$M, 2015–2019, NIH and NSF funding from Star Metrics
star_pctl	Double	starpp percentile among clusters
meth_fr	Double	Fraction of documents identified as method, 1996–2019
meth_pctl	Double	meth percentile among clusters
disc_fr	Double	Fraction of documents identified as discovery, 1996–2019
disc_pctl	Double	disc percentile among clusters
rev_fr	Double	Fraction of documents identified as review, 2015–2019
rev_pctl	Double	rev percentile among documents
trl_fr	Double	Fraction of documents identified as clinical trial, 2015–2019
trl_pctl	Double	trl percentile among clusters
nauth2	Integer	Number of authors with at least 2 papers in cluster, 2015–2019
nauth5	Integer	Number of authors with at least 5 papers in cluster, 2015–2019
age	Double	Mean age of papers in cluster
vit19	Double	Mean vitality of papers in cluster as of end-2019
3yrgrw	Double	Annualized growth rate in cluster from 2016–2019

**Table 5:** Data types for records in the TRANSP Excel sheet.

Index	Format	Description
PM5	Integer	PM5 cluster number
npoa	Integer	Number of open access documents per PubMed Central (PMCOA)
oa_fr	Double	Fraction of documents in cluster from PMCOA
coi_fr	Double	Fraction of PMCOA documents with a COI statement
coi_pctl	Double	coi percentile among clusters
fund_fr	Double	Fraction of PMCOA documents with a funding statement
fund_pctl	Double	fund percentile among clusters
reg_fr	Double	Fraction of PMCOA documents with a registration statement
reg_pctl	Double	reg percentile among clusters
data_fr	Double	Fraction of PMCOA documents with a data sharing statement
data_pctl	Double	data percentile among clusters
code_fr	Double	Fraction of PMCOA documents with a code sharing statement
code_pctl	Double	code percentile among clusters

The COUNT sheet contains a separate field for each year, 1996–2019, with integer document counts by cluster and year. The PHRASE, IDIO, MESH, ASJC and JNL sheets all have a similar format, an example of which is given in Table 6. The AUTH table is similar to these, but also contains a cpp (cites per paper) field to reflect relative author impact within the cluster. Tables 7–9 contain descriptions of the QUERY, CORE/REVIEW and METHDISC sheets, respectively.

**Table 6:** Common format for PHRASE, IDIO, MESH, ASJC and JNL Excel sheets.

Index	Format	Description
PM5	Integer	PM5 cluster number
rank	Integer	Field rank within cluster
descriptor	String	Phrase/idio/MeSH heading/category/journal
score	Double	Score or count of descriptor

**Table 7:** Data types for records in the QUERY Excel sheet.

Index	Format	Description
PM5	Integer	PM5 cluster number
#CORD	Integer	Number of documents found in the CORD-19 (Allen AI Covid 19) dataset
%CORD	Double	Fraction of documents found in the CORD-19 (Allen AI Covid 19) dataset
MICH	Integer	Number of documents with a University of Michigan address, 2015–2019
STAN	Integer	Number of documents with a Stanford University address, 2015–2019

**Table 8:** Data types for records in the CORE and REVIEW Excel sheets.

Index	Format	Description
PM5	Integer	PM5 cluster number
rank	Integer	Core paper rank
score	Double	Relative score based on relatedness values within cluster
type	String	Document type(s) from PubMed
source	String	Source metadata - PMID, title, journal, volume, page, year, DOI
ncited	Integer	Number of times cited from OCC, January 2020

**Table 9:** Data types for records in the METHDISC Excel sheet.

Index	Format	Description
PM5	Integer	PM5 cluster number
PMID	Integer	PubMed ID for document
method	String	identified as a method paper (=METH)
discovery	String	identified as a discovery paper (=DISC)

Updating of the PubMed model requires updating of all the databases that feed the model along with assignment of new papers to existing clusters, consideration of the formation of new clusters, recalculation of metrics, etc. We anticipate updating the model annually.

### Tableau workbook

Due to the variety of metadata and indicators associated with each cluster in the PM5 model, exploration of topics could proceed in many ways. To accommodate this potential, data from the Excel file (with the exception of the JNL_EXCL and METHDISC sheets) were incorporated into a Tableau workbook. Only the free software, Tableau Reader (https://www.tableau.com/products/reader), is required to interact with the workbook. Three data views have been constructed in Tableau – a map view, a scatterplot view, and a cluster detail view.

Filters (sliders and dropdowns) can be used to limit the clusters in the map and scatterplot views to those that meet a set of desired parameters. Each slider is tied to an indicator; thus one can filter based on variables such as impact (e.g., cites per paper, RCR), the relative presence of different types of documents (e.g., discovery, method, review, clinical trial), funding (e.g., dollars per paper from NIH/NSF, number of funding types per paper), and clinical application (e.g., research level, translational potential). Dropdowns include journal categories and MeSH terms which can be selected from menus or typed in (with available choices automatically reducing as text is entered). The display will be limited to clusters matching those choices. There is also an institution dropdown which limits the clusters to those in which a given institution has published in the 2015–2019 time period (see Table 7). This allows users to focus on clusters in which an institution of interest already has a publishing presence. Clusters can be sized using different parameters (e.g., number of papers, number of authors with at least 5 papers from 2015–2019, fraction of discovery papers) and can be colored using many of the features as well.

**Fig. 4:**
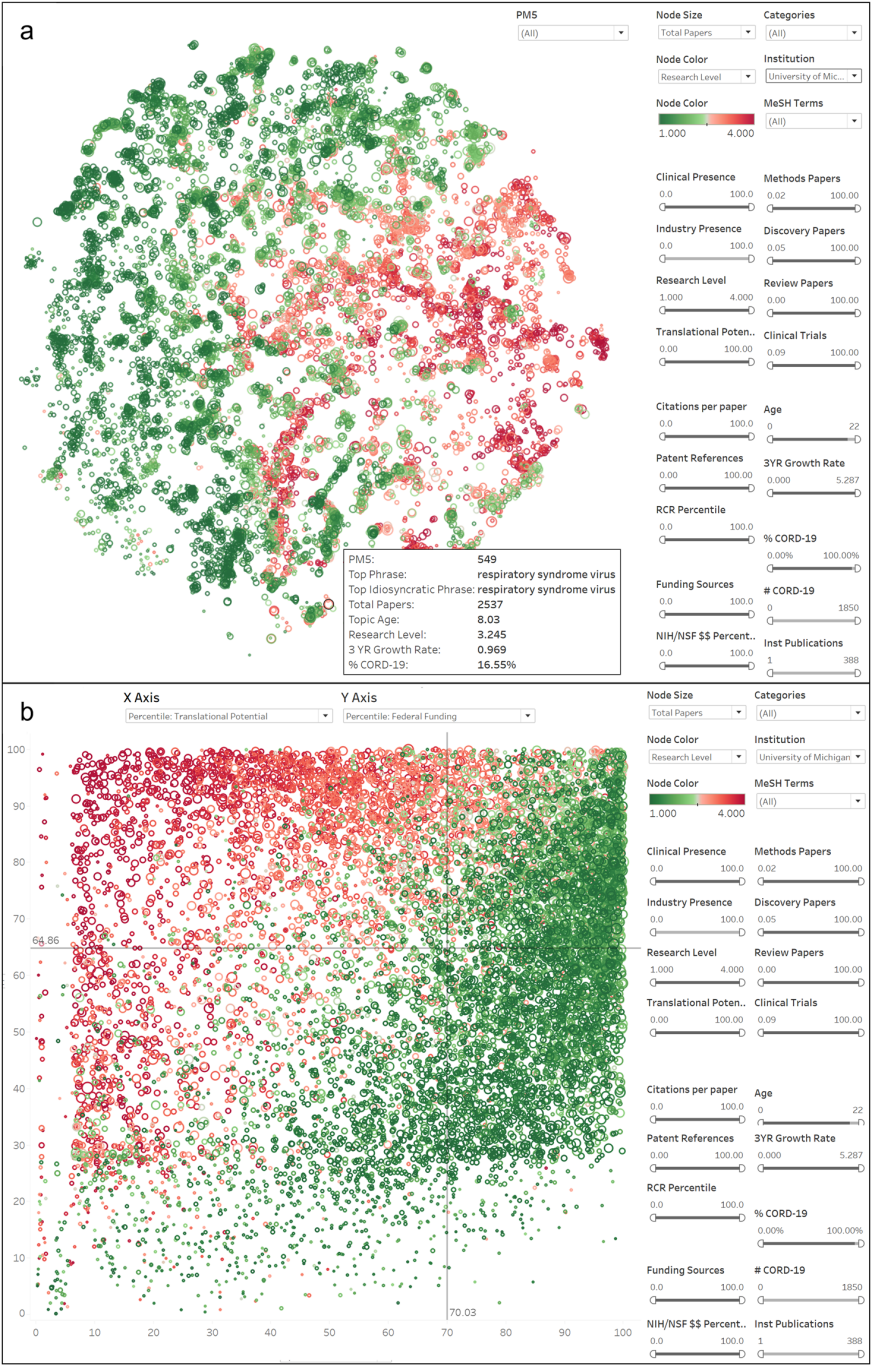
Tableau views of the PubMed model filtered to show only those clusters with UMMS papers. Color reflects the research level of each cluster. (**a**) Map view. (**b**) Scatterplot view with the approximate potential to translate percentile on the x-axis and NIH/NSF funding percentile on the y-axis.

Figure 4 shows map and scatterplot views of the clusters in which the University of Michigan has published in the 2015–2019 time period. Cluster coloring is based on research level, where dark red is the most basic and dark green is the most applied. An overview of cluster characteristics is shown when the mouse is used to hover over a cluster. Clicking on a cluster opens a cluster detail view that is equivalent to the Excel PM5_SHEET view (see Fig. 3).

The scatterplot view in Fig. 4b shows the relationship between translational potential (x-axis) and NIH/NSF funding (y-axis). The horizontal and vertical lines in the middle of the chart show medians, thus dividing the graph into four quadrants to enable quadrant-based analysis. In this view, basic research clusters tend to appear in the upper left quadrant while applied research clusters tend to appear at the lower right. The upper right quadrant is interesting in that it contains a mix of basic and applied content, although it is more heavily weighted toward applied.

### PMID to cluster listing

In addition to the Excel and Tableau workbooks, we make available the listing of PMID to PM5 cluster assignments in a separate tab separated (TSV) file. This enables linking of other PubMed-based data sources such as PKG^14^ to our model through PMID.

## Technical Validation

The validity of the model rests on the validity of the process used to create it. We start by acknowledging that there is no single clustering of a large-scale dataset that can be proven to be the most accurate. There are no ground truth data that can be used to determine the absolute accuracy of the clusters formed from a set of over 18 million documents. Nevertheless, there are ways to compare the results of different methodologies in a relative way that suggest that the clusters are coherent and useful.

Recently, a principled approach to comparing cluster solutions was introduced. It employs granularity-accuracy plots where cluster solutions are compared using their cluster size distributions (from which granularity is calculated) and pairwise relatedness data (from which relative accuracy is calculated)^4^. The most reliable results are obtained when the relatedness data used as the basis of comparison are independent of the relatedness measures used in clustering. It is also wise to use multiple bases of comparison where possible.

Prior to creating this PubMed model, we ran a large-scale experiment in which we compared seven relatedness measures, two citation-based, one text-based, and four hybrids using a set of nine million documents from PubMed^16^. The Leiden algorithm was used for each clustering run and each solution had roughly 20,000 clusters. Three different bases of comparison were used to determine the relative accuracies of the seven cluster solutions. One was based on the concentration of references of nearly 30,000 papers with large numbers of references within clusters. This measure is clearly biased toward citation-based relatedness measures. The second was based on the fraction of the top 20 SA scores in the entire set that were preserved within clusters. This measure is clearly biased toward text-based relatedness measures. The third was to calculate the fraction of papers with a common grant that were preserved within clusters. This measure is independent of both citation and text-based measures. Using a composite based on these three different relative accuracy measures, we found that the hybrid relatedness measure based on a 50:50 mix of direct citation and SA scores had the highest performance^16^. This hybrid solution preserved nearly 51% of the overall relatedness signal within clusters. These values suggest clusters of high quality given that there were nearly 20,000 clusters in the solution and also suggests that the PubMed model presented here is of similar quality in that the same process and relatedness measure were used.

To place this result in context, we note that other recent studies have similar findings regarding some specifics related to our model. Many studies at both small and large scale have found that hybrid relatedness measures produce better clusters than measures based solely on citations or text^3,32–37^. In addition, the principled approach for comparing cluster solutions has been used for multiple large-scale studies^3,18,38,39^ and is becoming a standard in the area of science mapping.

## Usage Notes

The recent COVID-19 pandemic is not only affecting individuals in negative ways, but it is also affecting research institutions. Although it is creating some new opportunities for COVID-19-related research, it is threatening many current research programs and structures. Under conditions of lockdown and social distancing, many labs have had to close. Some will re-open without issue, some will face uncertain conditions upon re-opening, while others may not be able to open at all. Funding availability will likely decrease, not only from agencies but also from local and regional governments that rely on decreasing tax revenues associated with economic downturn. For medical schools with associated hospitals, clinical margin revenue (a portion of which is used to fund research) is also greatly reduced. This very real scenario is being faced by universities across the world. How can universities balance their fiduciary duties to the financial health of the organization while simultaneously supporting the research mission from within a contracting financial system? Our PubMed model provides a quantitative view of the research landscape that facilitates informed decision-making.

We provide here example workflows to show how the Tableau tool can be used. The examples show how one medical school (in this case the University of Michigan Medical School, UMMS) could navigate the current landscape, first by looking for opportunities related to historical literature associated with coronavirus, and then by looking at topics related to a cohort of African American and Native American researchers.

Since early 2020, the Semantic Scholar team at the Allen Institute for AI, along with other partners, has been compiling a set of literature related to coronavirus – the COVID-19 Open Research Dataset (CORD-19) – and has made it publicly available for use by researchers^40^. The May 31, 2020 version of this dataset contained 139,952 documents among which 98,228 unique PMID could be identified. Of these, 67,452 PMID were from 1996 through 2019 and were found in our PubMed model. CORD-19 contains literature associated not only with coronavirus (e.g., CoV, SARS, MERS) but since mid-May also contains literature associated with co-morbidity factors and related medical conditions (respiratory problems, etc.) It also contains over 30,000 documents from 2020, many of which have been recently added to PubMed from preprint servers such as medRxiv and bioRxiv despite not yet having completed the peer review process. These documents from 2020 are not in our model but will be added in the future.

**Fig. 5:**
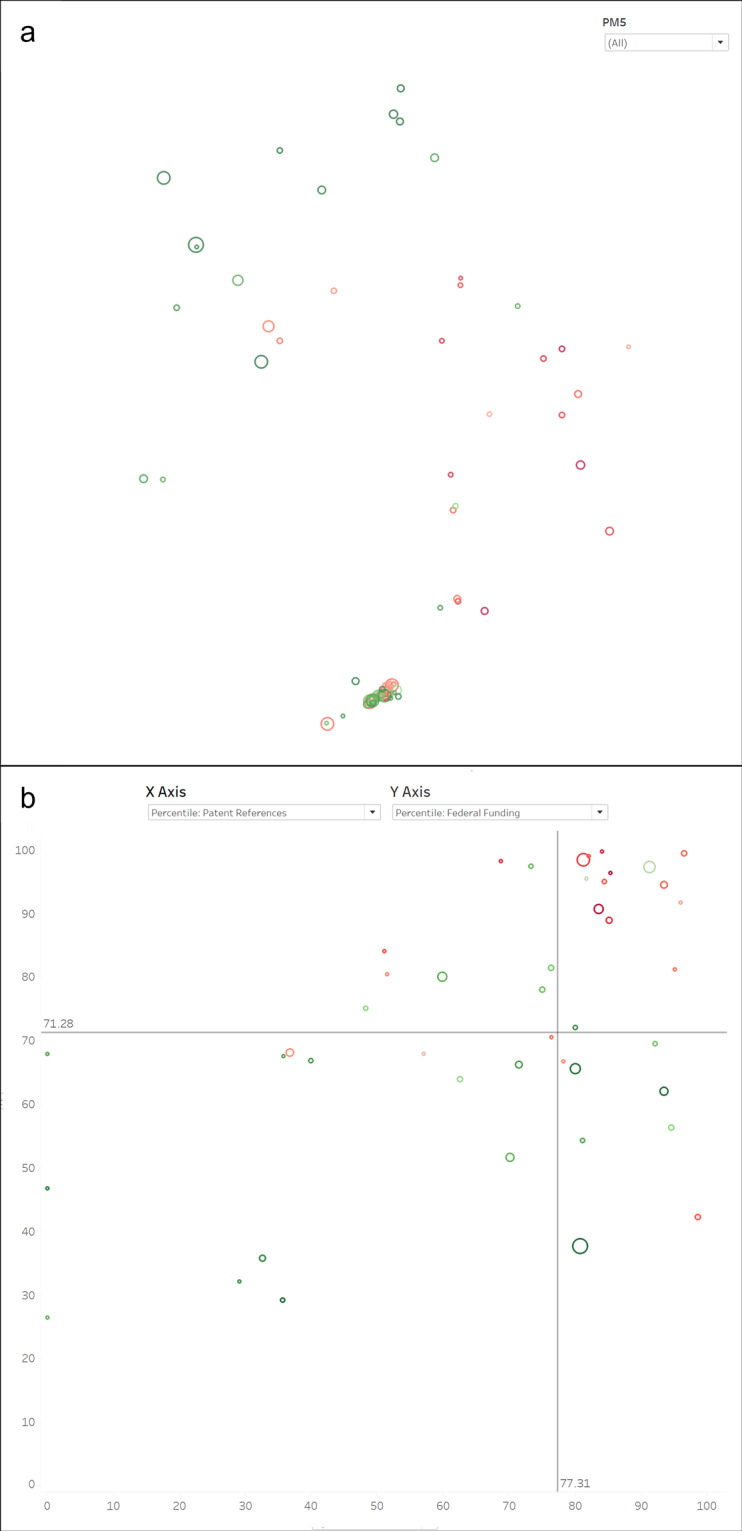
Tableau views of subsets of clusters related to coronavirus. (**a**) Map view of clusters with at least 25 CORD-19 documents and a CORD-19 document concentration of at least 10%. (**b**) Scatterplot view of clusters further filtered to those containing UMMS papers.

Figure 5a shows the location of 85 clusters that contain at least 25 documents from CORD-19 and where the concentration of CORD-19 documents is at least 10%. Clusters are sized by the number of CORD-19 documents and colored using research level (red = basic, green = applied). These are the clusters that are most related to coronavirus as defined by CORD-19. A majority of the CORD-19 clusters are at the bottom of the map in an area focused around viruses known to affect the respiratory system (e.g., influenza, SARS, MERS, etc.) Clusters in other areas of the map focus on a variety of topics such as other viruses, proteins, and treatments or devices that have recently become associated with treatment of COVID-19 (e.g., cerebral oxygenation, mechanical thrombectomy).

This view of the coronavirus-related literature is quite different from others that have been published recently^41,42^ and separates the literature into distinct topics much more precisely. For example, Colavizza *et al*.^41^ recently characterized and mapped nearly 40k publications from the April 4, 2020 version of CORD-19. Using topic modeling they identified 15 topics, the largest of which is labeled ‘public health and epidemics’ and contains roughly 20% of the corpus. Citation-based clustering was also done, resulting in 23 clusters with at least 100 documents and a much larger number of very small clusters. The largest cluster contained 7,300 documents with a focus on coronaviruses and related molecular biology analyses. In contrast, our solution has two separate clusters for SARS (one related to the virus and the other to infection) along with separate clusters on MERS, human coronaviruses, equine coronaviruses, bat viruses, and many other different types of viruses. Both strategic and tactical decision making are thus better enabled using the type of granular classification of documents available in our model than in other related mapping exercises.

Figure 5b further limits to only those clusters in which UMMS has published from 2015–2019. Identification of the UMMS papers was not done using the Tableau application but were obtained from a query to PubMed. Once the list of UMMS documents was obtained, clusters were identified for each PMID, the number of documents was counted by PM5 cluster, and these numbers were then imported into the Tableau file and included in the filtering and labeling capabilities. The identification of UMMS-authored documents could have also been done by simply extracting a list of PMID from the institution’s research information management system (e.g., https://experts.umich.edu/), a task that any institution with such a system could accomplish with minimal effort.

In Fig. 5b, clusters are sized by the number of UMMS papers to show relevance to UMMS and are plotted as a function of funding percentile (y-axis) and patent reference percentile (x-axis). If UMMS were to choose to prioritize activities in which a) they have a strong publication presence, b) historical U.S. funding levels are high, and c) commercial potential exists, large clusters at the upper right of this graph might be prioritized. In this case, the top three candidates (largest three clusters in the upper right quadrant) are focused on murine norovirus infections, rhinovirus infections, and RNA viruses. Despite being represented in the CORD-19 dataset, none of these clusters has the type of overt relationship to COVID-19 that would suggest that immediate short-term funding could be obtained. However, one of the smaller clusters in that quadrant, #12391 on ribosomal frameshifting, could be a candidate for such funding in that frameshifting could play a role in mutation and was already investigated for SARS years ago. Also, the largest cluster in the upper left quadrant is clearly important in the current climate, #2673 on the effects of school closures and social distancing on epidemics.

As COVID-related research efforts continue to increase in both scale and scope, universities must not forget about other areas of research that, while not directly related to the current health crisis, are nonetheless vitally important to the health of a nation. For instance, it is important to support the research activities of groups that are currently under-represented, such as female and minority researchers.

UMMS has a substantial number of African-American/black (AA/B) and Native American (NA) researchers. Between 2010 and 2018, these individuals submitted 657 research proposals to external funding sources and published 4,489 papers from 1996–2019 that were indexed in PubMed. Of these, 3,995 appear in our model; most of the remainder are physics and chemistry papers in journals that we removed from the model. Note that this analysis requires that data from the university be linked to the model and shows how the model can be easily extended to include such data to facilitate advanced analysis.

Publication activities of AA/B and NA researchers at UMMS are consistent with the most recent in-depth study of racial bias at NIH. Hoppe et al. studied over 157,000 R01 applications to characterize how racial bias affects AA/B researchers during different stages of their career. They provided compelling evidence that the topic mix of R01 applications from AA/B researchers is very different from that of White researchers, and that AA/B applicants tend to propose research on topics with lower award rates^43^. They noted that AA/B applications were far more likely to involve human subjects than those from White applicants. AA/B applications tended to focus on health disparities such as AIDS and patient-focused interventions. In contrast, clusters associated with osteoarthritis, cartilage, prion, corneal, skin, iron, and neuron contained no applications from AA/B researchers.

**Fig. 6:**
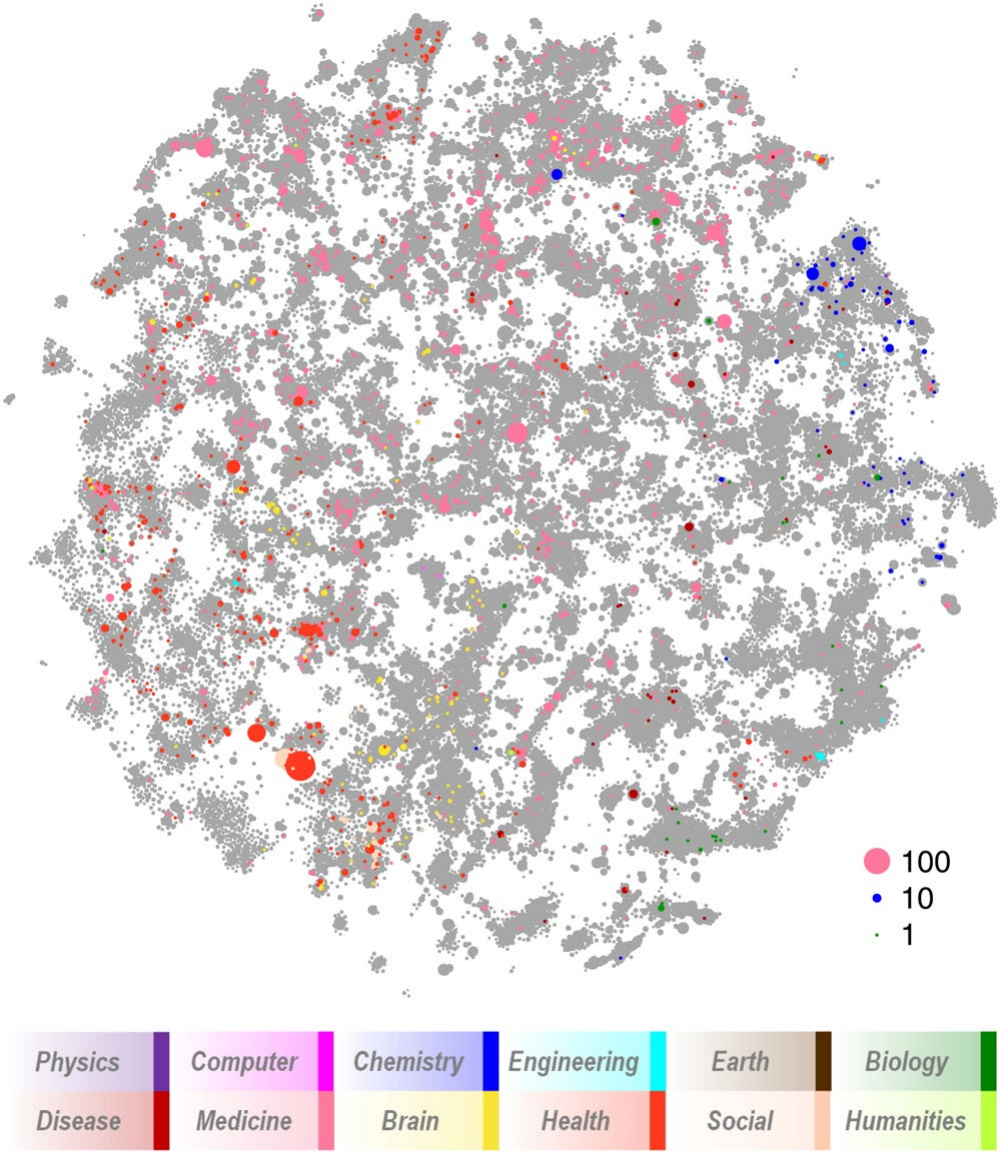
Publication profile of African American and Native American principal investigators at UMMS overlaid on the PubMed map. Sizes of colored circles reflect numbers of publications.

The 3,995 papers published by AA/B and NA researchers at UMMS are shown in Fig. 6 as colored circles overlaid on a gray version of the map. The distribution of publications over the map is notably unbalanced with relatively few in the lower right quadrant (which is dominated by biology and infectious disease) and much higher in the lower left quadrant in areas that reflect racial disparities. For example, the topic in which AA/B and NA researchers at UMMS have the largest number of publications is #6953 which focuses on physical health, mental health and economic disparities in minority populations.

Figure 6 suggests that the topic choices of AA/B and NA researchers at UMMS are skewed in a way that is consistent with NIH findings on AA/B R01 applications. By extension, given that these topics are, on the whole, less well-funded by NIH than other topics, this suggests that minority researchers are disadvantaged in a fundamental way. It behooves universities to be aware of the topic choices of their researchers, how these topics and researchers might be disadvantaged, and then to use this knowledge to inform equity focused interventions.

We note that recently published PubMed Knowledge Graph^14^ contains complementary document level information such as extracted bioentities, disambiguated authors and institutions that could be added to the PubMed model by linking through PubMed IDs to facilitate additional types of analysis.

## Data Availability

The Leiden algorithm was used for clustering and is freely available at https://github.com/vtraag/leidenalg.
